# Ionic thermal up-diffusion in nanofluidic salinity-gradient energy harvesting

**DOI:** 10.1093/nsr/nwz106

**Published:** 2019-07-30

**Authors:** Rui Long, Zhengfei Kuang, Zhichun Liu, Wei Liu

**Affiliations:** School of Energy and Power Engineering, Huazhong University of Science and Technology, Wuhan 430074, China

**Keywords:** energy harvesting, ionic thermal up-diffusion, ionic voltage source, nanofluidics

## Abstract

Advances in nanofabrication and materials science give a boost to the research in nanofluidic energy harvesting. Contrary to previous efforts on isothermal conditions, here a study on asymmetric temperature dependence in nanofluidic power generation is conducted. Results are somewhat counterintuitive. A negative temperature difference can significantly improve the membrane potential due to the impact of ionic thermal up-diffusion that promotes the selectivity and suppresses the ion-concentration polarization, especially at the low-concentration side, which results in dramatically enhanced electric power. A positive temperature difference lowers the membrane potential due to the impact of ionic thermal down-diffusion, although it promotes the diffusion current induced by decreased electrical resistance. Originating from the compromise of the temperature-impacted membrane potential and diffusion current, a positive temperature difference enhances the power at low transmembrane-concentration intensities and hinders the power for high transmembrane-concentration intensities. Based on the system's temperature response, we have proposed a simple and efficient way to fabricate tunable ionic voltage sources and enhance salinity-gradient energy conversion based on small nanoscale biochannels and mimetic nanochannels. These findings reveal the importance of a long-overlooked element—temperature—in nanofluidic energy harvesting and provide insights for the optimization and fabrication of high-performance nanofluidic power devices.

## INTRODUCTION

Recently, nanofluidic salinity-gradient energy harvesting via ion channels or membranes has drawn increasing concerns due to the advances in materials science and nanotechnology [1–3], which could offer much higher power density than the macro reverse electrodialysis systems [4–10]. Guo *et al.* [11] obtained a maximum power output of 26 pW in a single nanopore and claimed that, by adopting parallel nanopore arrays, power density can be enhanced by one to three orders over previous ion-exchange membranes, indicating its potential to harvest the blue energy (about 1.4–2.6 TW) released by mixing seawater and river water [12] and enhance the power extracted for membrane-based osmotic heat engines [13]. The performance of the nanofluidic energy-conversion system mainly relies on channel geometry, surface-charge density, ion types and temperature that impact the electric double layer (EDL) overlapping degree and the ion-transportation characteristics [4,14–27]. Lin *et al.* [28] achieved a power of up to 120 pW via a mesoscopic conical pore modified with poly-L-lysine. Cao *et al.* [29] investigated the impacts of nanopore length on the nanofluidic reversed electrodialysis (RED) system and found that, at short nanopore length, the system demonstrates an anomalous, non-Ohmic response due to degraded charge selectivity and induced strong-ion-concentration polarization. Zhang *et al.* [30] revealed that, using slippery nanopores, the energy efficiency at the short nanopore length could be dramatically increased under large salt concentrations. Moreover, the power-density gap between single-pore and membrane-based nanofluidic systems is also discussed [27]. The power density on multi-pore membranes could not be simply linearly scaled, due to significant concentration polarization.

Previous efforts focusing on the nanofluidic energy-conversion system mainly deal with the isothermal reservoirs [31–35], where the membrane potential *E_mem_* reads

(1) \begin{equation*} {E}_{mem}=\left(2{t}_{+}-1\right)\frac{RT}{zF}\ln \left(\frac{\gamma_H{C}_H}{\gamma_L{C}_L}\right) \end{equation*}

Here, *t_+_* is the cation-transfer number, *C_i_* is the concentration and *Y_i_* is the activity coefficient of the bulk concentrations. *T* is the solution temperature. The conventional viewpoint suggests that improving the membrane potential requires a larger temperature and a long channel length to guarantee a large selectivity and a high effective concentration difference [29,34]. This intuitive judgement accounts for increasing temperature to achieve better performance, which originates from the results based on isothermal conditions [36]. However, the asymmetric temperatures of the solution reservoirs are a very important yet long-overlooked element that impacts the performance of the nanofluidic devices [7,37]. With transmembrane temperature difference applied, due to the Soret effects, ions diffuse along or opposite to the temperature gradient, stemming from their different thermal responsiveness [38–40]. Although the Soret effect may be ignorable in nanofluidic ion rectification and energy conversion [41–43], the ion-transportation characteristics are significantly impacted by the temperature-dependent physical properties [34,36].

**Figure 1.**
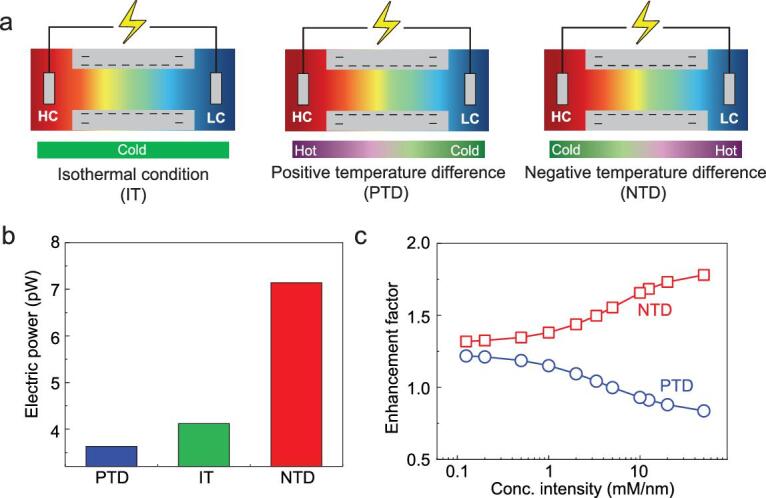
Anomalous temperature dependence in the nanofluidic energy-harvesting system. (a) Schematic illustration of the ion-concentration profiles with varied transmembrane temperature differences. The left side is of high concentration (HC) with temperature *T_L_* and the right side is of low concentration (LC) with temperature *T_R_*. IT, PTD and NTD represent the isothermal conditions (*T_L_* = *T_R_*), positive temperature difference (*T_L_* > *T_R_*) and negative temperature difference (*T_L_* < *T_R_*). (b) Electrical power under different temperature differences. A negative temperature difference can significantly improve the power output due to suppressed ICP and improved selectivity. The concentration difference is 1000-fold; the channel length is 50 nm. IT, PTD and NTD refer to *T_L_* = *T_R_* = 298 K, *T_L_* = 318 K and *T_R_* = 298 K, and *T_L_* = 298 K and *T_R_* = 318 K. The concentration difference is 1000-fold; the channel length is 50 nm. (c) At small transmembrane-concentration intensities, both positive and negative temperature differences contribute to the electrical power. At high transmembrane-concentration intensities, a negative temperature difference promotes the power extraction and a positive temperature difference hinders the power extracted. Here, the enhancement factor is defined as the power under the IT condition divided by that under the NTD/PTD. In the calculation, the transmembrane-concentration intensity is calculated by varying the channel length from 20 to 8000 nm with a fixed concentration difference (1000-fold).

In this context, we deal with the temperature-dependent performance of nanofluidic energy-conversion systems based on thermodynamic analysis and the numerical-simulation method. In contrast to the previous results that focused on isothermal conditions, a negative temperature difference can obviously improve the membrane potential due to the impact of ionic thermal up-diffusion (enhanced ion diffusion along the osmotic direction) that promotes the selectivity and suppresses the ion-concentration polarization (ICP), especially at the low-concentration (LC) side, which results in dramatically enhanced electric power (Fig. 1). A positive temperature difference lowers the membrane potential due to the impact of ionic thermal down-diffusion (weakened ion diffusion along the osmotic direction), although it promotes the diffusion current induced by decreased electrical resistance. Originating from the compromise of the temperature-impacted membrane potential and diffusion current, the system presents an anomalous temperature dependence at different transmembrane-concentration intensities. More intriguingly, at a low transmembrane-concentration intensity and high concentration difference, the membrane potential stays constant, which is only determined by the temperature at the LC side for equalized temperature in the significant EDL-overlapping zone and the salt reservoir. And the optimal transmembrane-concentration intensity at maximum power shifts left and right according to the direction of the temperature gradient while the value of the maximum power remains unchanged. These findings reveal the importance of the long-overlooked element—the transmembrane temperature difference—in nanofluidic energy conversion. We can easily fabricate tunable ionic voltage sources, where the voltage can be tuned by the LC temperature and the internal resistance is impacted by the transmembrane temperature differences. And waste heat can be employed to enhance the power output by adjusting the transmembrane temperature difference to use the nanoscale biochannels and mimetic nanochannels: establishing a negative temperature difference to increase the optimal transmembrane-concentration intensity to match the high transmembrane-concentration intensity under that small-membrane scale, thus guaranteeing a larger power extracted and a high ionic flux.

## RESULTS AND DISCUSSION

In the current study, the nanofluidic energy-conversion process was analysed under the thermodynamic-diffusion theory and numerical calculation [44] (Supporting Information). Compared to the experimental study, we can calculate separately the cation-contributed and the anion-contributed currents based on the Poisson–Nernst–Planck equations, Navier-Stokes equations, as well as energy-conservation equations. Here, we consider a cylindrical nanopore with radius *R_n_* = 10 nm [34], varied length *L_n_* and a constant surface-charge density of −0.05 C/m^2^ in a solid membrane that contacts with two similar large reservoirs at different salt concentrations and temperatures, respectively (Supplementary Fig. 1). The transmembrane-concentration difference ranges from 10-fold to 1000-fold by varying the high-concentration (HC) (*C_H_*) side and fixing the LC (*C_L_*) side to 1 mM. The temperatures of the reservoirs range from 298 to 318 K [36]. Actually, the surface-charge density is impacted by the solution properties and temperature. The relation of the temperature and the surface-charge density is fairly complex. Therefore, the assumption of constant surface-charge density is employed here [41,42]. Performances with varied surface-charge densities are analysed in the Supporting Information. The membrane potential *E_mem_* is calculated via linear interpolation from different electrical currents under varied applied voltages (Supporting Information). The maximum power is calculated using *P_max_* = *I_osm_ E_mem_*/4. *I_osm_* is the short-circuit current (diffusion current). The relevant theoretical analysis and the validity of the numerical method can be found in the Supporting Information and references therein.

**Figure 2.**
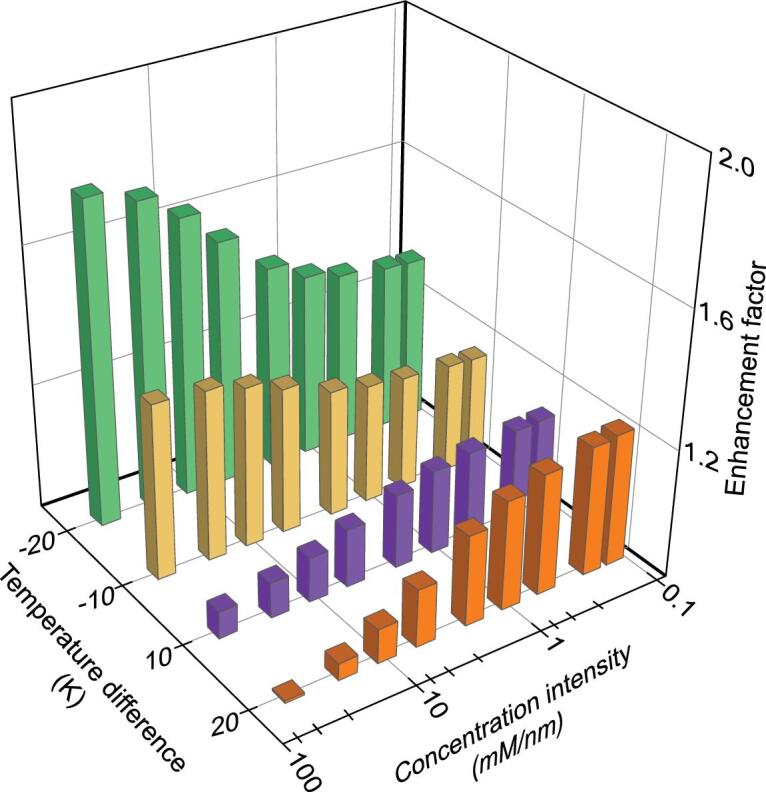
Anomalous temperature dependence under varied transmembrane-concentration intensities. A negative temperature difference can significantly improve the electrical power while the behavior of the extracted power under the positive temperature difference exhibits strong dependence of the transmembrane-concentration intensity. At low transmembrane-concentration intensities, a positive temperature difference contributes to the power output, which decreases the power at high transmembrane-concentration intensities. And a larger positive temperature difference leads to an augmented power enhancement. At larger transmembrane-concentration intensities, a positive temperature difference hinders the power output, especially with larger temperature differences. In the calculation, the transmembrane-concentration intensity is calculated by varying the channel length from 20 to 8000 nm with a fixed concentration difference (1000-fold).

As the concentration profile does not vary linearly across the nanopore due to ICP, the driven force for ion diffusion, the concentration gradient, could not be directly calculated as the transmembrane-concentration difference divided by the nanopore length. Generally, at a given transmembrane-concentration difference, a short nanopore length leads to augmented ion diffusion. Here, we employ a parameter, transmembrane-concentration intensity, to qualitatively illustrate the strength of the driven force for ion diffusion, which is defined as the transmembrane-concentration difference divided by the nanopore length. Larger nanopore length leads to smaller transmembrane-concentration intensity and weakened ion diffusion, and vice versa. We systematically investigate the temperature dependence of the nanofluidic power-generation system under various transmembrane-concentration intensities and temperature differences. A negative temperature difference can significantly improve the electrical power while the behavior of the extracted power under the positive temperature difference exhibits strong dependence of the transmembrane-concentration intensities (Fig. 2). At low transmembrane-concentration intensities, a positive temperature difference contributes to the power output and, more obviously, at larger temperature differences. At high transmembrane-concentration intensities, the electrical power is hindered by the positive temperature difference. Furthermore, the electrical power first increases with increasing transmembrane-concentration intensities, reaches its maximum value, then decreases (Supplementary Figs 5 and 6). Here, the optimal transmembrane-concentration intensity can be carefully tuned by applying asymmetric temperature differences, which shifts left under positive temperate differences and right under negative temperature differences (Supplementary Fig. 6). More intriguingly, at a given concentration difference, the maximum power remains unchanged for varied reservoir temperatures at the HC side when the reservoir temperature at the LC side is fixed (Supplementary Fig. 6).

**Figure 3.**
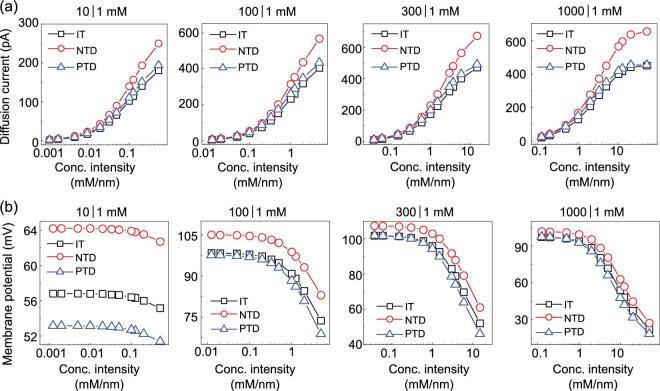
Diffusion current (a) and membrane potential (b) versus transmembrane-concentration intensities at different temperature differences, where IT, PTD and NTD represent the isothermal conditions (*T_L_* = *T_R_* = 298 K), positive temperature difference (*T_L_* = 318 K, *T_R_* = 298 K) and negative temperature difference (*T_L_* = 298 K, *T_R_* = 318 K). The transmembrane-concentration intensity is tuned by varying the channel length from 20 to 8000 nm at a fixed concentration difference, which ranges from 10- to 1000-fold. The diffusion current increases with increasing transmembrane-concentration intensities under varied temperature differences. Both positive and negative temperature differences are beneficial for the diffusion current, but the latter impacts very obviously. The membrane potential reaches a plateau at low transmembrane-concentration intensities. A negative temperature difference contributes to the membrane potential and a positive temperature difference degrades the membrane potential. At high concentration differences and low transmembrane-concentration intensities, the membrane potential remains constant, which is only determined by the temperature at the low-concentration side.

**Figure 4.**
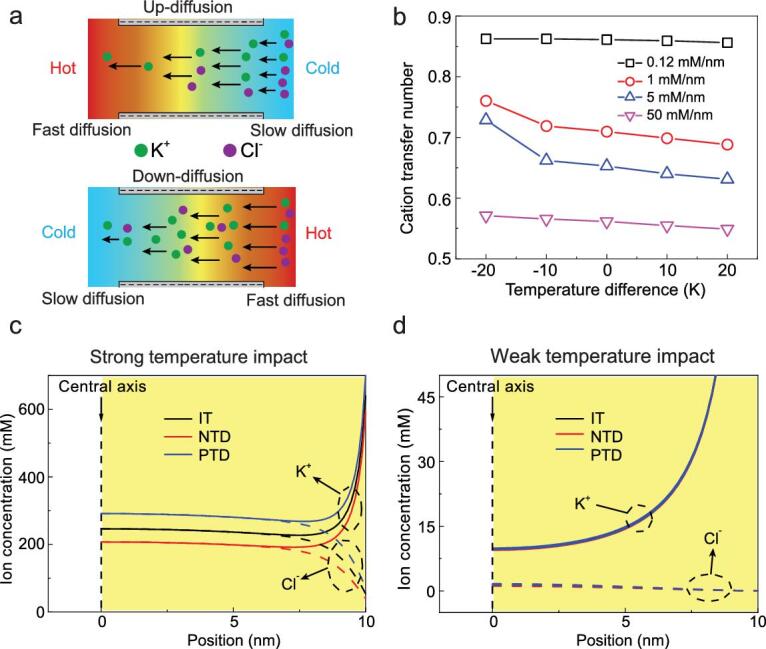
(a) Schematic illustration of the ionic thermal up-diffusion and down-diffusion in the nanofluidic energy-conversion system. (b) The cation-transfer number under varied temperature differences. (c) and (d) Radius-concentration profiles for cations and anions at a cross-section at the low-concentration (LC) exit. At a high transmembrane-concentration intensity (short channel with length at 20 nm), the ion concentration at the LC exit is strongly impacted by the transmembrane temperature difference. A negative temperature difference decreases the ion concentrations, leading to an enhanced EDL-overlapping degree and improved selectivity. A positive temperature difference contributes to the ion concentrations, resulting in a worsened EDL-overlapping degree and degraded selectivity. At a low transmembrane-concentration intensity (long channel with length at 5000 nm), the cation/anion-concentration profile presents no obvious difference under various temperature differences. In the calculation, the concentration difference is 1000-fold.

**Figure 5.**
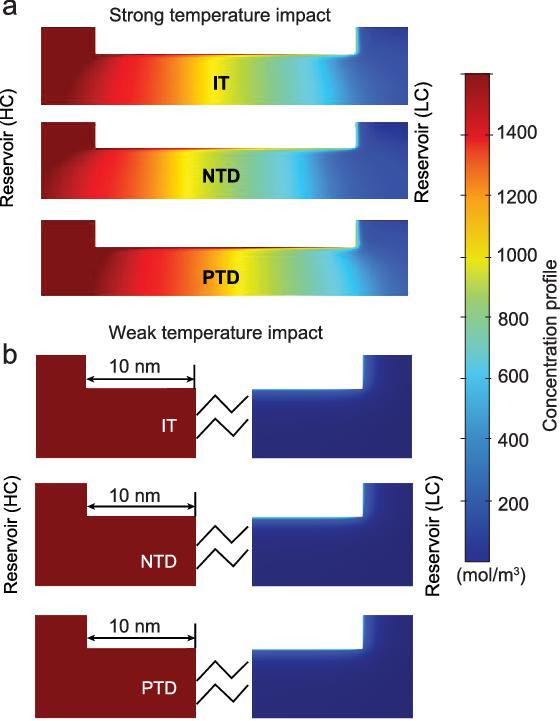
Temperature-impacted ion-concentration polarization. (a) and (b) Concentration profiles (C_K_^+^ + C_Cl_^−^) of high and low transmembrane-concentration intensities under varied temperature differences. At a large transmembrane-concentration intensity (short channel with length at 20 nm), the ICP is significantly impacted by the applied asymmetric temperatures. At a small transmembrane-concentration intensity (long channel with length at 5000 nm), the ICP presents no obvious difference under various temperature differences. The concentration difference is 1000-fold.

We further investigate the diffusion current and the membrane potential as functions of temperature differences under varied transmembrane-concentration intensities (Fig. 3). At any given channel length and transmembrane-concentration intensity, the electric resistance is mainly determined by the average temperature of the salty solution (Supplementary Fig. 7). Both positive and negative temperature differences are beneficial for the diffusion current due to the enhanced ion conductivity and decreased electrical resistance. As the ionic current induced by the Soret effect is rather weak compared to that induced by ion diffusion [41–43], the negative temperature difference always enhances the ionic current due to augmented ionic-diffusion coefficients at elevated temperatures. As shown in Fig. 3, the membrane potential is sensitive to the temperature difference, very prominently at higher transmembrane-concentration intensities. A negative temperature difference contributes to the membrane potential while a positive temperature difference goes against the membrane potential. Here, a counterintuitive phenomenon occurs that, at high concentration differences and long channel lengths, the membrane potential is determined only by the temperature at the LC side and is independent of the temperature at the HC side (Supplementary Fig. 11).

Here, we employ the terminology ‘ionic thermal up-diffusion' to give a better illustration of the asymmetric temperature dependence, which means the osmotic diffusion is enhanced along the osmotic-diffusion direction due to the temperature impact (Fig. 4a). On the contrary, ‘ionic thermal down-diffusion' means that the osmotic diffusion is weakened along the osmotic-diffusion direction. The EDL thickness can be represented by the Debye length $\lambda$, which is impacted by the ion concentration, $\lambda \propto 1/\sqrt{C}$, where *C* is the local concentration. Larger concentration leads to a lower value for the Debye length and a weakened EDL-overlapping degree. With a negative temperature difference applied, the diffusion coefficient increases along the diffusion direction. The ion diffusion is enhanced by the ionic thermal up-diffusion and ion concentrations are reduced in the nanochannel, which contributes to the EDL overlapping, leading to improved selectivity (Fig. 4b and Supplementary Fig. 10). When a positive temperature is applied, ion concentrations are augmented in the nanochannel, resulting in worsened EDL overlapping and a degraded ion selectivity. At high transmembrane-concentration intensities, strong ICP exists. The drop in the membrane potential with increasing transmembrane-concentration intensity mainly originates from the degraded selectivity and worsened ICP (Fig. 5a). Under the negative temperature difference, due to the impact of ionic thermal up-diffusion, the concentration at the LC exit is decreased, resulting in suppressed ICP (Fig. 4c). The concentration at the LC exit under the positive temperature difference is increased due to ionic thermal down-diffusion, inducing worsened ICP. Due to improved selectivity and suppressed ICP, the membrane potential is augmented under the negative temperature difference. On the other hand, a positive temperature difference reduces the membrane potential for degraded selectivity and worsened ICP.

**Figure 6.**
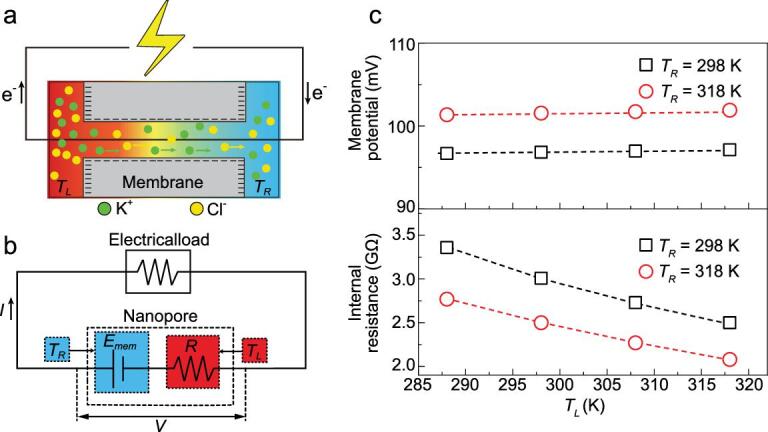
(a) and (b) Schematic graph of the tunable ionic voltage source at low transmembrane-concentration intensity and high concentration difference by applying asymmetric temperatures. (c) Membrane potential and internal resistance under asymmetric temperatures. For a given *T_R_*, the membrane potential remains unchanged at various *T_L_*. The internal resistance decreases with increasing *T_L_*. The voltage is controlled by adjusting the LC-reservoir temperature, and the internal resistance is tuned by changing the HC-reservoir temperature. In the calculation, the concentration difference is 1000-fold and the channel length is 5000 nm.

At lower transmembrane-concentration intensities, the impact of the EDL-overlapping degree is very significant (Supplementary Fig. 10). The cation-transfer number exhibits a slight difference under varied temperature differences (Fig. 4b and Supplementary Fig. 8). Besides, the ICP is hindered and does not present obvious differences under varied temperature differences (Figs 4d and 5b and Supplementary Fig. 12). Hence, the membrane potential is mainly determined by the temperature in the significant EDL-overlapping section. For LC difference, significant EDL overlapping exists in the entire channel and the membrane potential differs under varied temperature differences. For high concentration differences, significant EDL overlapping only exists near the exit section (Supplementary Fig. 10). A long channel length weakens the transmembrane temperature gradient and equalizes the solution temperature in the effective channel section and that of the LC reservoir. Furthermore, the Seebeck coefficient of the KCl solution is about 35 μV/K [45]. A temperature difference of 20 K leads to a potential variation of 0.7 mV, which is very small compared to the calculated membrane potential. The impact of the Seebeck effect can be neglected and the membrane potential is determined by the concentration-gradient-induced potential. In this situation, the membrane potential is only determined by the temperature at the LC side. Here, a tunable ionic voltage source can be established at a long channel and an high concentration difference with asymmetric temperatures (Fig. 6). A larger average transmembrane temperature difference decreases the internal resistance while the membrane potential stays unchanged. The voltage is controlled by adjusting the LC-reservoir temperature, and the internal resistance is tuned by changing the HC-reservoir temperature.

Based on the above considerations, a negative temperature difference can significantly improve the membrane potential, and the diffusion current, bringing an obvious augmentation to the electrical power. Originating from the compromise of the temperature-impacted membrane potential and the diffusion current, a positive temperature difference contributes to electrical power at low transmembrane-concentration intensities and hinders the electrical power at large transmembrane-concentration intensities.

**Figure 7.**
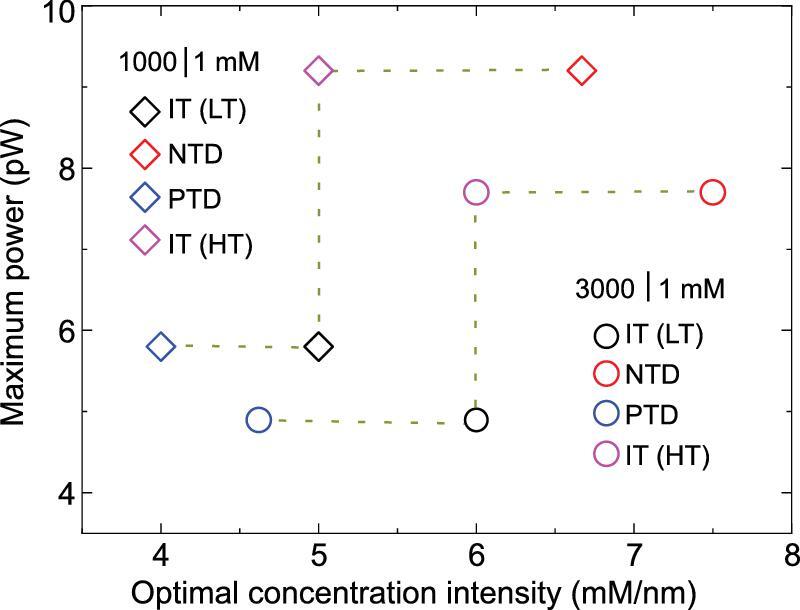
Temperature-impacted transmembrane-concentration intensity at maximum power. Due to the equalized temperature in the significant EDL-overlapping zone and the LC salt reservoir, the maximum power remains unchanged and is determined by the temperature of the LC reservoir. As the negative temperature differences suppress the ICP and improve selectivity, the balance point between the polarization-dominated zone and the resistance-dominated zone shifts to the short channel length, leading to a high optimal transmembrane-concentration intensity. On the contrary, a positive temperature difference worsens the ICP and degrades the selectivity; it takes a longer channel to balance the polarization and the resistance impacts, resulting in a decreased optimal transmembrane-concentration intensity. Here, IT, PTD and NTD represent the isothermal conditions (*T_L_* = *T_R_* = 298 K for low temperature (LT) and *T_L_* = *T_R_* = 318 K for high temperature (HT)), positive temperature difference (*T_L_* = 318 K, *T_R_* = 298 K) and negative temperature difference (*T_L_* = 298 K, *T_R_* = 318 K).

Furthermore, the energy-conversion efficiency with asymmetric temperatures applied is deduced as (Supporting Information):

(2) \begin{equation*} \eta =\frac{\left(2{t}_{+}-1\right)\frac{E_{mem}}{2}}{\frac{R}{F}\ln \frac{\alpha_H^{T_H}}{\alpha_L^{T_L}}+\frac{\Delta {\mu^{\Theta}|}_{T_L}^{T_H}}{F}} \end{equation*}

where ${\Delta {\mu_i}^{\Theta}|}_{T_L}^{T_H}$ is the difference in the chemical potential at the standard state at temperatures *T_H_* and *T_L_*. Enhanced power output and decreased input Gibbs free energy under the negative temperature difference contribute to the energy-conversion efficiency. The positive temperature difference decreases the membrane potential, deteriorates the ion selectivity and augments the Gibbs free energy consumed, resulting in lowered energy-conversion efficiency. Given the concentration difference of 1000-fold and channel length of 50 nm, the energy-conversion efficiency is augmented by 37.3% under the negative temperature difference, and is decreased by 32.2% under the positive temperature difference (Supplementary Fig. 16). If the heat needed to establish the transmembrane temperature difference is considered, the energy efficiency will be significantly decreased due to huge transmembrane heat loss. Therefore, we can use waste heat to construct a negative transmembrane temperature difference, and thus improve its efficiency [46].

As additional remarks, the nanometer-thick, single-pore or porous materials may not be suitable for nanofluidic energy conversion due to the intrinsic small scale, which corresponds to the ICP-dominated region [47,48]. To efficiently use these small-scale materials for energy extraction, waste heat could be used to adjust the transmembrane temperature difference. We can form a negative temperature difference to increase the optimal transmembrane-concentration intensity to match the high transmembrane-concentration intensity under that membrane scale, thus to enhance the power extracted and provide a high ionic flux (Fig. 7). Furthermore, the maximum power under a given concentration difference and LC-reservoir temperature does not vary with the HC-reservoir temperature, making it promising to fabricate nanofluidic power stacks with differently sized membranes for stable energy output.

## CONCLUSION

In conclusion, we investigate the asymmetric temperature dependence in the process of the nanofluidic energy-conversion system. Results are somewhat counterintuitive compared to the conventional viewpoints obtained under the isothermal conditions. A negative temperature difference can significantly improve the membrane potential due to the impact of ionic thermal up-diffusion that promotes the selectivity and suppresses the ICP, especially at the LC side, which results in dramatically enhanced electrical power. A positive temperature difference lowers the membrane potential due to the impact of ionic thermal down-diffusion, although it promotes the diffusion current induced by decreased electrical resistance. The electrical power exhibits anomalous behavior under the positive temperature difference, originating from the compromise in the membrane potential and diffusion current impacted by ionic thermal down-diffusion. Finally, we have proposed a simple and efficient way to fabricate tunable ionic voltage sources and enhance the power out by adjusting the transmembrane temperature difference. Understanding of the anomalous temperature dependence provides insight for the optimization and fabrication of high-performance nanofluidic power devices.

## Supplementary Material

nwz106_Supplemental_File
