# Supplementary material for: Ionic thermal up-diffusion in nanofluidic salinity-gradient energy harvesting
Source: Natl Sci Rev. 2019 Jul 30;6(6):1266–73. doi: 10.1093/nsr/nwz106 (PMC8291421; doi:10.1093/nsr/nwz106)
Supplement: nwz106_Supplemental_File [file nwz106_supplemental_file.pdf]

Supporting Information for

Ionic thermal up-diffusion in nanofluidic salinity gradient energy harvesting

Rui Long,* Zhengfei Kuang, Zhichun Liu, and Wei Liu*

School of energy and power engineering, Huazhong University of Science and Technology, Wuhan 430074, P. R. China.

* Correspondence authors r_long@hust.edu.cn (R. Long), w_liu@hust.edu.cn (W. Liu)

Table of the contents

1. Thermodynamic analysis
2. Numerical simulation
3. Check of mesh independence
4. Validation by the experimental data
5. Calculation of the membrane potential
6. Electric power
7. Electrical resistance of nanochannels
8. Charge selectivity of long and short nanochannels
9. Concentration profiles at long channel lengths
10. Membrane potential
11. Ion concentration polarization
12. Impacts of surface charge density
13. Impacts of viscous dissipation
14. Energy efficiency
15. References

1. Thermodynamics analysis

1.1 Chemical potential at the standard state at different temperatures

For any ideal gas, the chemical potential can be written as

$$\mu(T,P) = h(T,P) - T \bullet s(T,P) \tag{S1}$$

where h and s are the enthalpy and entropy, respectively. The specific heat at constant pressure can be written as

$$Cp = (\frac{\partial h}{\partial T})_P = (\frac{\partial h}{\partial s})_P \bullet (\frac{\partial s}{\partial T})_P = T(\frac{\partial s}{\partial T})_P \tag{S2}$$

Furthermore, according the Maxwell relation , we have

$$(\frac{\partial s}{\partial P})_T = -(\frac{\partial v}{\partial T})_P = -\frac{R}{P} \tag{S3}$$

The derivative of the entropy is

$$ds = (\frac{\partial s}{\partial P})_T dP + (\frac{\partial s}{\partial T})_P dT = Cpd\ln T - R\ln P \tag{S4}$$

On the other hand, the chemical potential can be written as a function of the chemical potential at the standard state $\mu^{\Theta}(T)$, which is only determined by the temperature.

$$\mu(T,P) = \mu^{\Theta}(T) + RT\ln P \tag{S5}$$

Hence we have

$$\mu^{\Theta}(T) = h(T_0,P) + \int_{T_0}^{T} CpdT - T\int_{T_0}^{T} Cpd\ln T - T \bullet s(T_0,1) \tag{S6}$$

where $(T_0,1)$ is a reference state.

Therefore the difference of the chemical potential at the standard state at different temperatures (T_1 and T_2)

$$\Delta\mu^{\Theta}|_{T_1}^{T_2} = \mu^{\Theta}(T_2) - \mu^{\Theta}(T_1) \tag{S7}$$

1.2 Energy conversion efficiency at maximum power condition

The energy conversion efficiency at maximum power condition can be derived based on the thermodynamic diffusion theory. Considering the ion diffusion from the high concentration solution at concentration C_H and temperature T_H to the low concentration solution at concentration C_L and temperature T_L. At the steady state, the flux of Gibbs free energy consumed is [1]

$$\Delta\dot{G} = \Delta\dot{G}_H + \Delta\dot{G}_L = (\mu_{+,H} - \mu_{+,L})\dot{n}_{+,H} + (\mu_{-,H} - \mu_{-,L})\dot{n}_{-,H} \tag{S8}$$

where + and - denotes the cation (K^+) and anion (Cl^-) respectively. μ and $\dot{n}$ are chemical potential and ion flux, given by[2]

$$\mu_{i,j} = \mu_{i,0}(T_j) + RT_j \ln \alpha_j \tag{S9}$$

$$\dot{n}_{i,H} = -\frac{|I_i|}{F} \tag{S10}$$

where I_i is the current contributed by the cation (i is +) or anion (i is –). j denotes high concentration (H) or low concentration (L).

Therefore the flux of Gibbs free energy consumption is

$$\Delta G = \sum_i (RT_H \ln \alpha_{i,H} - RT_L \ln \alpha_{i,L} + \Delta\mu_i^{\Theta} |_{T_L}^{T_H}) \frac{I_i}{F} \tag{S11}$$

The power achieves its maximum value $P_{\max} = (|I_+| - |I_-|)V$, at $V = E_{mem}/2$. The energy efficiency at maximum power is defined as the ratio of the retrievable electric power to the Gibbs free energy of mixing

$$\eta = \frac{(|I_+| - |I_-|)\frac{E_{mem}}{2}}{\frac{R}{F}\ln\frac{\alpha_{+,H}{}^{T_H}}{\alpha_{+,L}{}^{T_L}}|I_+| + \frac{R}{F}\ln\frac{\alpha_{-,H}{}^{T_H}}{\alpha_{-,L}{}^{T_L}}|I_-| + \frac{|I_+|}{F}\Delta\mu_+^{\Theta}|_{T_L}^{T_H} + \frac{|I_-|}{F}\Delta\mu_-^{\Theta}|_{T_L}^{T_H}} \tag{S12}$$

Applying $\frac{\alpha_{+,H}{}^{T_H}}{\alpha_{+,L}{}^{T_L}} \cong \frac{\alpha_{-,H}{}^{T_H}}{\alpha_{-,L}{}^{T_L}} \cong \frac{\alpha_H{}^{T_H}}{\alpha_L{}^{T_L}}$, $\Delta\mu_+^{\Theta}|_{T_L}^{T_H} \cong \Delta\mu_-^{\Theta}|_{T_L}^{T_H}$ and $|I_+|/|I_-| \cong t_+/(1-t_+)$ yields to the simplified expression for maximum power efficiency

$$\eta = \frac{(2t_+ - 1)\frac{E_{mem}}{2}}{\frac{R}{F}\ln\frac{\alpha_H{}^{T_H}}{\alpha_L{}^{T_L}} + \frac{\Delta\mu^{\Theta}|_{T_L}^{T_H}}{F}} \tag{S13}$$

Under isothermal conditions ($T_H = T_L = T$), Equation (S13) returns to

$$\eta = \frac{(2t_+ - 1)\frac{E_{mem}}{2}}{\frac{RT}{F}\ln\frac{\alpha_H}{\alpha_L}} \tag{S14}$$

Equation (S14) is exactly the maximum power efficiency obtained in previous literatures under iso thermal conditons [3].

Furthermore, if the heat needed to establish the transmembrane temperature difference considered, the heat transferred from the high temperature side to the low temperature side

through heat conduction and convection is denoted as Q_{trans}. Therefore the energy efficiency considering the tranmembrane heat transfer is

$$\eta = \frac{(|I_+| - |I_-|)\frac{E_{mem}}{2}}{(|I_+| + |I_-|)\frac{R}{F}\ln\frac{\alpha_H{}^{T_H}}{\alpha_L{}^{T_L}} + (|I_+| + |I_-|)\frac{\Delta\mu^{\Theta}|_{T_L}^{T_H}}{F} + Q_{trans}} \tag{S15}$$

2. Numerical simulation

As depicted in Figure S1, we consider a cylindrical nanopore with radius R_n = 10 nm and length L_n in a solid membrane, which contacts with two same large reservoirs of radius R_r =1000 nm and length L_r = 1000 nm at salt concentrations C_H and C_L (C_H>C_L), and temperatures T_L and T_R, respectively. The continuum-based model coupling Poisson-Nernst-Planck equations, Navier-Stokes equations and energy conservation equation is employed to illustrate the electrostatics, ionic mass transport, heat transfer and energy transportation characteristics [4,5]

$$-\nabla\bullet(\varepsilon\nabla\phi)=F\sum_{i=1}^{2}z_i c_i \tag{S16}$$

$$\nabla\bullet\boldsymbol{J}_i=0\text{, where }\boldsymbol{J}_i=c_i\boldsymbol{u}-D_i\nabla c_i-\frac{D_i z_i F c_i}{RT}\nabla\phi-\frac{2D_i\alpha_i c_i}{T}\nabla T \tag{S17}$$

$$-\nabla p+\nabla\bullet(\mu\nabla\boldsymbol{u})-F\sum_{i=1}^{2}z_i c_i\nabla\phi-\frac{1}{2}|\boldsymbol{E}|^2\nabla\varepsilon=0 \tag{S18}$$

$$\nabla\bullet(\rho\boldsymbol{u})=0 \tag{S19}$$

$$\rho C_p\boldsymbol{u}\bullet\nabla T=\nabla\bullet(k\nabla T) \tag{S20}$$

where ϕ is the electrical potential. $\boldsymbol{J}_i$, c_i, D_i and z_i are the ionic flux, concentration, diffusivity, and valence of the *i*th ionic species, respectively (i =1 for K^+ and i =2 for Cl^-). α_i is the reduced Soret coefficient, which is defined as $\alpha=TS_T/2$, where S_T represents the intrinsic Soret coefficients of cations or anions in the absence of charge coupling[4]. α = 0.5 for K^+ and α = 0.1 for Cl^- [6]. F, R and T are the Faraday constant, universal gas constant and the fluid temperature. ε, p, and $\boldsymbol{u}$ are the permittivity, pressure, and velocity of the fluid. ρ, C_p ,and k are the density, specific capacity, and thermal conductivity. $\boldsymbol{E}=-\nabla\phi$ is the electric field. As presented in Equation (S17), the ionic flux consists of the convective, ionic diffusive, electrodiffusive and thermodifusive fluxes, respectively corresponding to the first, second, third and last terms in the right hand. In Equation (S18), the electrostatic force and the elecrothermal force (dielectric force) are also considered to include the impact of the temperature gradient across the nanopore. At lower applied electric fields, the effect of viscous dissipation was unable to generate a local temperature gradient, and the influence of viscous dissipation could be neglected [7]. Therefore we neglect the viscous dissipation in the energy conservation equation in present study. Further justification can be found in Figure S15.

All the properties for solving the above equations are temperature-dependent. The properties of the salt solutions are treated as the same with that of the water. The temperature dependence of relative permittivity (ε_r) is [8,9]

$$\varepsilon_r = \exp(4.47615 - 4.60128\times10^{-3}\Delta T + 2.6952\times10^{-7}(\Delta T)^2) \quad \text{(S21)}$$

where $\Delta T = T - 273.15$, $0 \le \Delta T \le 100$.

The temperature dependence of the viscosity is [10]

$$\mu = 2.414\times10^{-5}\times10^{247.8/(T-140)},\ 273.15 \le T \le 643.15 \quad \text{(S22)}$$

Based on the Nernst-Haskell equation, the diffusive coefficient D_i (i =1 for K^+ and i =2 for Cl^-) [10,11]

$$D_i = \frac{RT}{F^2}\left[\frac{1/|z_i|}{1/\lambda_i^0}\right] \quad \text{(S23)}$$

where the λ_i^0 is the limiting conductance of the ith ionic species. The limitting conductances of K^+ and Cl^- are listed below

$$\lambda_1^0 = 40.5017 + 1.2194(T - 273.15) + 0.0041859(T - 273.15)^2 \quad \text{(S24)}$$

$$\lambda_2^0 = 76.35 + 1.54037(T - 298.15) + 0.00465(T - 298.15)^2 - 0.00001285(T - 298.15)^3 \quad \text{(S25)}$$

To solve the above equations, proper boundary conditions are needed. The surface charge density of the membrane wall is $\sigma = -50mC/m^2$. And the reservoir wall is free of charge $\sigma = 0$. The wall of the nanopore and that of the reservoir are impermeable to ions. Ω_n are non-slip and Ω_r is slip, where Ω_n ,and Ω_r are the surface of the nanopore and the reservoir. The pressure at both the reservior ends is set to zero. Therefore, the following boundary conditions apply.

$$-\varepsilon \boldsymbol{n}\bullet\nabla\phi = 0 \text{ on } \Omega_r \text{ and } \Lambda_j \quad \text{(S26)}$$

$$-\varepsilon \boldsymbol{n}\bullet\nabla\phi = \sigma \text{ on } \Omega_n \quad \text{(S27)}$$

$$\boldsymbol{n}\bullet\boldsymbol{J}_i = 0 \text{ on } \Omega_r \text{ and } \Lambda_j \quad \text{(S28)}$$

$$T = T_j \text{ at } \Lambda_j \quad \text{(S29)}$$

where $\boldsymbol{n}$ is the normal vector Λ_j represents the reservoir end.

The electric current is calculated by

$$I = \int_\Lambda F(\sum_{i=1}^{2} z_i \boldsymbol{J}_i)\bullet\boldsymbol{n}d\Lambda \quad \text{(S30)}$$

The transference number (t_+) indicating the nanopore selectivity can be calculated as

$$t_{+} = \frac{I_1}{I_1 + |I_2|} \tag{S31}$$

where I_1 and I_2 are the electrical current stepping from the cation and anion, respectively.

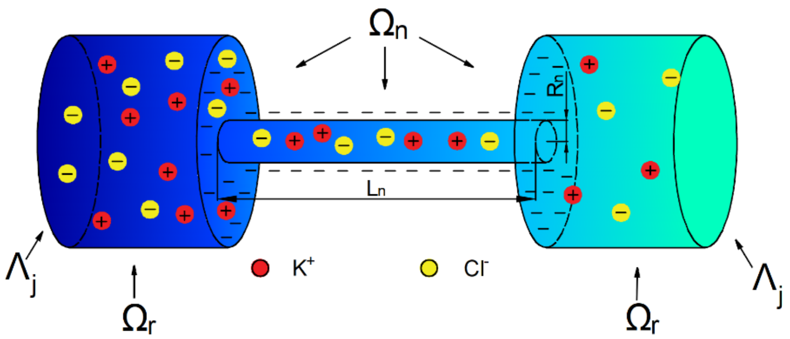

Figure S1. Schematic of the nanofluidic energy conversion system through a cylindrical nanopore.

3. Check of mesh independence

The calculation is conducted through the commercial multiphysics software COMSOL based on the finite element method with quadrilateral meshes. Here, structured mesh is employed in present study. The element size increases exponentially along the radius direction from the channel surface to the axial center to well capture the EDL region. The mesh numbers are adjusted under varied channel length. For example, at given channel length (L=1000 nm) and concentration difference (1000-fold), mesh numbers between ca. 131000 and ca. 407000 are used to confirm that calculation data are completely converged and mesh-independent, as shown in Figure S2. The osmotic current does not vary with increasing mesh numbers, when the mesh number is larger than 205000. Therefore, we choose a mesh number of ca. 205000 for calculating the ionic current for channel length L=1000 nm. For different channel lengths, varied mesh number are used to confirm that calculation data are completely converged and mesh independent.

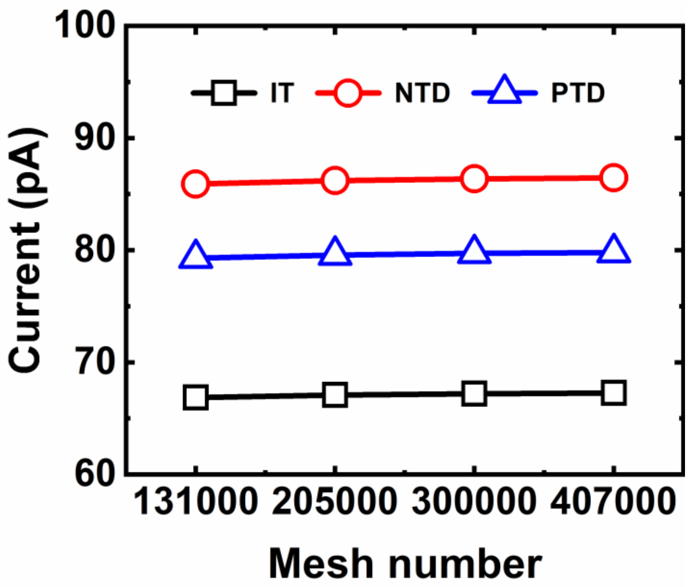

Figure S2. Osmotic current under different mesh numbers, where IT, PTD, and NTD represent the isothermal conditions (T_L=T_R=298K), positive temperature difference (T_L=318K, T_R=298K) and negative temperature difference (T_L=298K, T_R=318K). In the calculation, the channel length L=1000 nm, and the concentration difference is 1000-fold.

4. Validation by the experimental data

As there is no available experimental data under the conditions of asymmetric temperatures, here we only conducted a comparison between the present calculated data and the experimental data of the salinity gradient dependent osmotic current in a boron nitride (BN) nanotube with R_n = 40 nm and L_n = 1250 nm at pH = 5.5. from Siria et al.[12], which was obtained under the isothermal conditions. In Ref. [12], the surface charge density of BN is derived based on analytical expression for nanopore conductance, which neglects the influences of ICP, and electroosmotic and diffusioosmotic flows. In Ref. [13], the the surface charge density under pH = 5.5 is fitted to be -0.06 C/m^2. Therefore, we adopt the surface charge density of -0.06 C/m^2 to calculate the osmotic currents under various concentration ratios. The prediction from the present model is in very good agreement with the experimental data of Siria et al.[12], as shown in Figure S3. The error is the largest at high concentration ratios due to the fact the nanopore wall of BN nanotube is hydrodynamic slip which contributes to the ionic current. Therefore, the ionic current at high concentration ratios, is slightly lower than the experimental one.

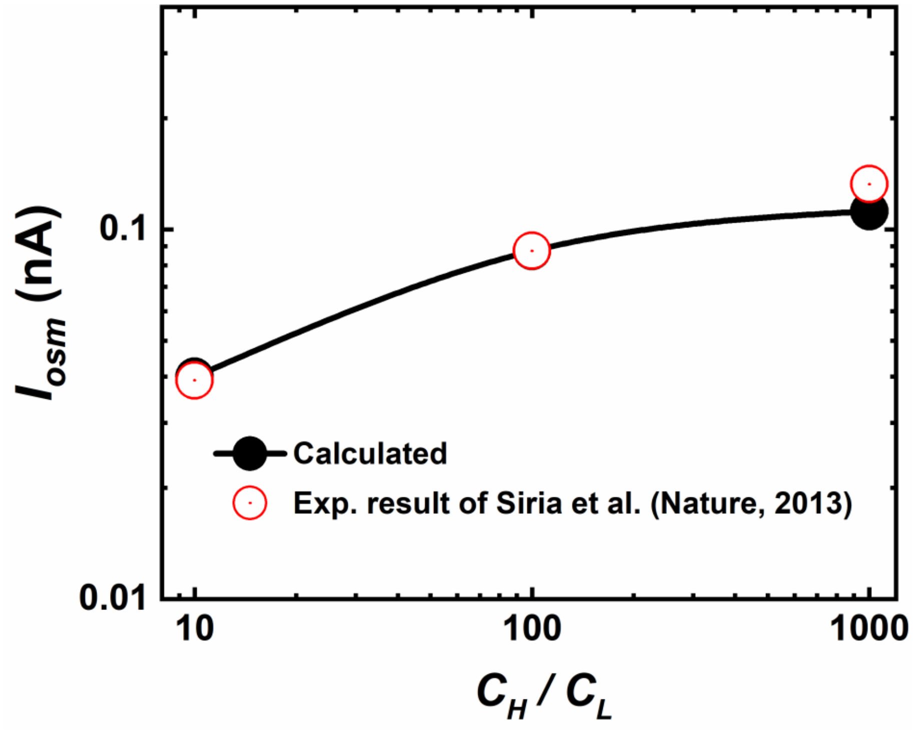

Figure S3. Comparison between present calculated data and the experimental data of the salinity gradient dependent osmotic current in a boron nitride nanotube with R_n = 40nm and L_n = 1250 nm at pH = 5.5. from Siria et al.[12]. The pH = 5.5 corresponds to the surface charge density of -0.06C/m^2 [13]. The prediction from the present model is in very good agreement with the experimental data of Siria et al.[12].

5. Calculation of the membrane potential

As shown in Figure S4, under the presence of asymmetric temperature differences, the voltage still presents a linear relationship with the electric current, indicating the system exhibits the Ohm behaviour. Therefore, we can fit the *I*-*V* curve (straight line) by calculating different electrical currents at varied applied voltages. The membrane potential E_{mem} is obtained through the intersection with the *V*-axis. That is to say, the membrane potential E_{mem} is calculated via linear interpolation from different electrical currents at varied applied voltages.

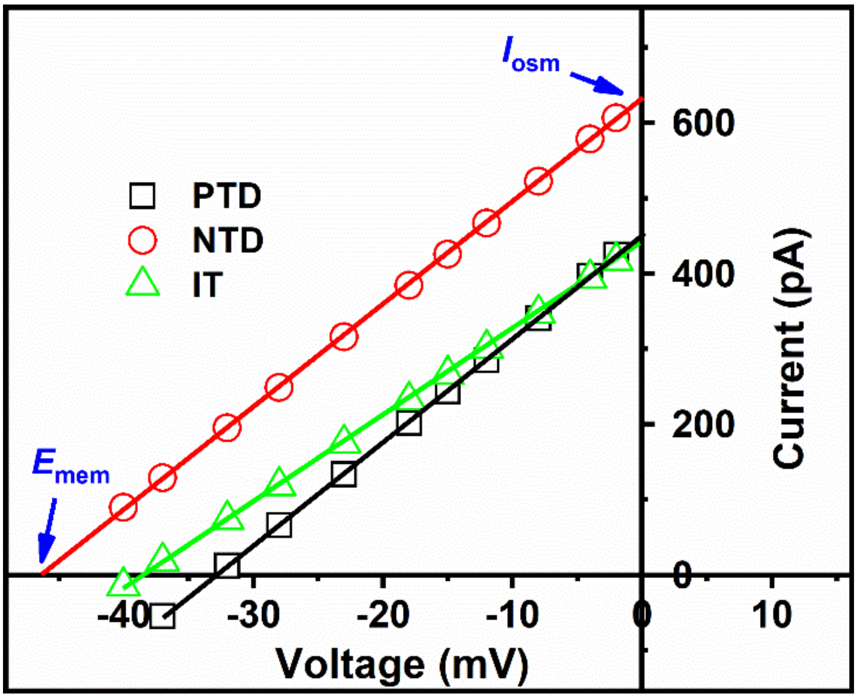

Figure S4 *I*-*V* curves under different temperature differences, where IT, PTD, and NTD represent the isothermal conditions (T_L=T_R=298 K), positive temperature difference (T_L=318 K, T_R=298 K) and negative temperature difference (T_L=298 K, T_R=318 K). In the calculation, the concentration difference is 1000-fold, the channel length is 50 nm.

6. Electric power

As the concentration profile does not vary linearly across the nanopore due to ion concentration polarization, the driven force for ion diffusion, concentration gradient, could not directly calculated as the transmembrane concentration difference divided by the nanopore length. Generally, at a given transmembrane concentration difference, short nanopore length leads to augmented ion diffusion. Here we employ a parameter, transmembrane concentration intensity, to qualitatively to illustrate the strength of driven force for ion diffusion, which is defined as the transmembrane concentration difference divided by the nanopore length. Larger nanopore length leads to smaller transmembrane concentration intensity and weakened ion diffusion, and vice versa. The electric power for asymmetric temperatures under different transmembrane concentration intensities is shown in Figure S5. At low transmembrane concentration intensities and concentration differences, the electric power increases monotonously. As the transmembrane concentration intensity and concentration difference increases further, the electric power first increases with increasing transmembrane concentration intensity, reaches its maximum value, then decreases.

Figure S6 shows the electric power vs transmembrane concentration intensities under different temperature differences at high concentration difference. There exits an optimal transmembrane concentration intensity corresponding to the maximum power, where the compromise between the selectivity, ICP and the electric resistance is established. For high concentration differences, significant EDL overlapping only exists near the exit section. Long channel length weakens the transmembrane temperature gradient and equalizes the solution temperature in the effective channel section and that of the LC reservoir. Therefore, the maximum power stays unchanged for varied reservoir temperatures at the high concentration side when the reservoir temperature at the LC side is fixed. The optimal concentration intensity is also presented in Figure 7 in the main text. As the negative temperature differences suppress the ICP and improves selectivity, the balance point between the polarization dominated zone and the resistance dominated zone shifts to the short channel length, leading to a high optimal transmembrane concentration intensity. On the contrary, a positive temperature difference worsens the ICP and degrades the selectivity, it takes a longer channel to balance the polarization and the resistance impacts, resulting in a decreased optimal transmembrane concentration intensity.

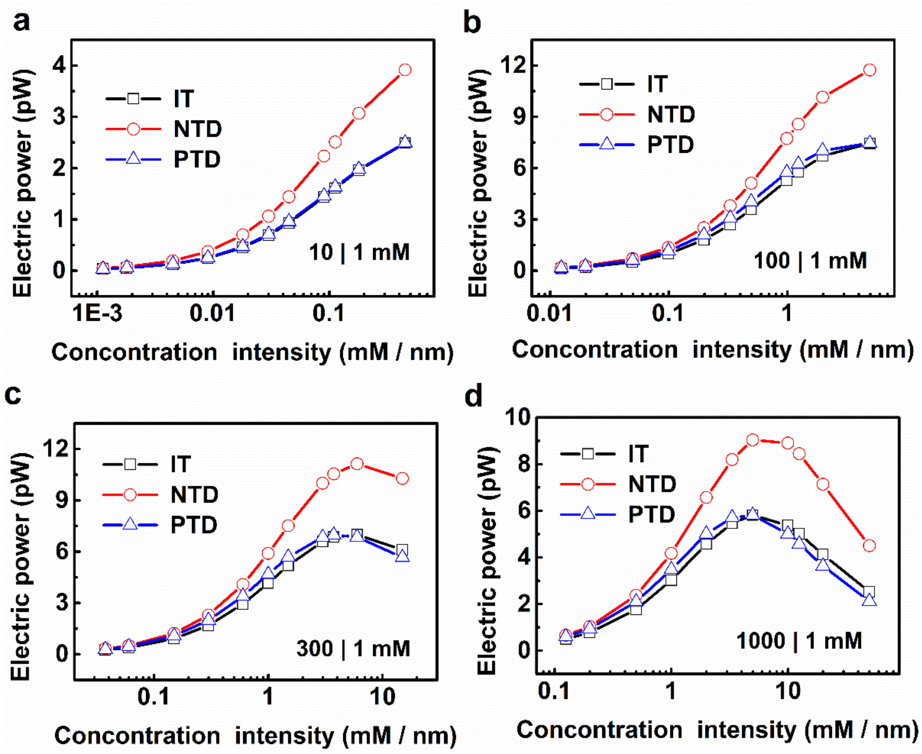

Figure S5. The electric power vs transmembrane concentration intensities under different temperature differences, where IT, PTD, and NTD represent the isothermal conditions (T_L=T_R=298 K), positive temperature conditions (T_L=318 K, T_R=298 K) and negative temperature conditions (T_L=298 K, T_R=318 K). (a) 10|1 mM, (b) 100|1 mM, (c) 300|1 mM, and (d) 1000|1 mM. The transmembrane concentration intensity is calculated as the concentration difference dividing the nanopore length.

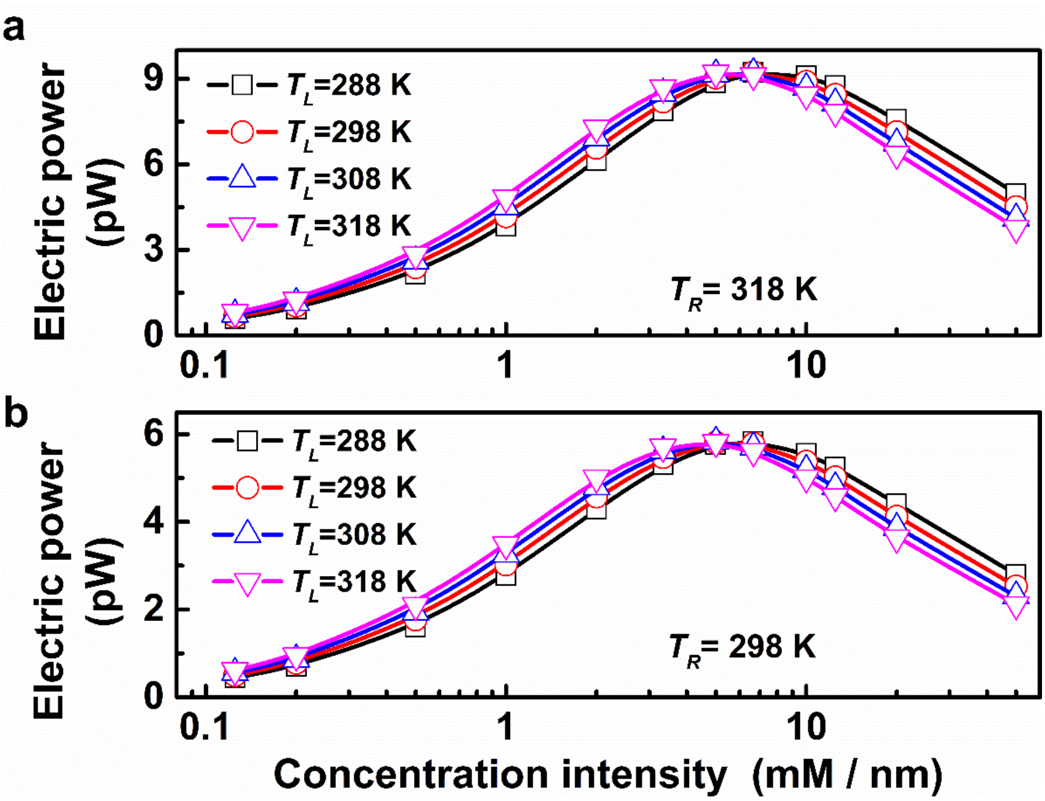

Figure S6. The electric power vs transmembrane concentration intensities under different temperature differences. In the calculation, the applied concertation difference is 1000-fold. And the transmembrane concentration intensity is calculated as the concentration difference dividing the nanopore length.

7. Electric resistance of nanochannels

The electric resistance of nanochannels under different temperature and concentration differences is shown in Figure S7. The electric resistance under the positive and negative temperature differences is decreased, induced by the increased average temperature of the salt solution. The curves for the negative temperature difference and the positive temperature difference almost coincide with each other, due to equal average solution temperature. For very short channels, the electric resistance under the negative temperature difference slightly derivates from that under the positive temperature difference due to strong ion diffusion and ion concentration polarization. Therefore, the temperature depended performance under the negative and positive temperature differences are mainly caused by the temperature impacted electromotive force and electric resistance.

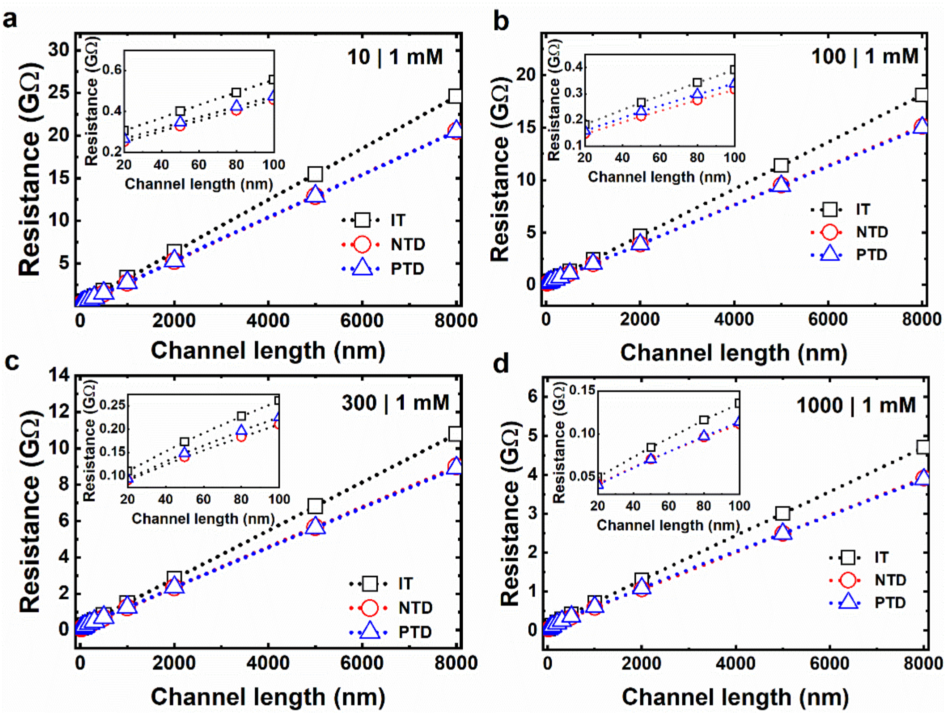

Figure S7. The electric resistance of the nanochannel length under varied temperature and concentration differences, where IT, PTD, and NTD represent the isothermal conditions (T_L=T_R=298 K), positive temperature difference (T_L=318 K, T_R=298 K) and negative temperature difference (T_L=298 K, T_R=318 K). (a) 10|1 mM, (b) 100|1 mM, (c) 300|1 mM, and (d) 1000|1 mM.

8. Charge selectivity of long and short nanochannels

The cation transfer number for varied transmembrane concentration intensities at different temperature differences are presented in Figure S8. A negative temperature difference improves the selectivity while a positive temperature difference goes against the selectivity. At lower transmembrane concentration intensities, the cation transfer number nearly stays unchanged at different temperature differences. As the transmembrane concentration intensity increases, the cation transfer number decreases. A negative temperature contributes to the ion selectivity and a positive temperature difference degrades the ion selectivity. The concentration difference significantly affects the ion selectivity of the nanochannels under strong and weak temperature impact conditions (Figure S9). Under strong temperature impact condition, the cation transfer number decreases much dramatically with increasing concentration difference. And the temperature difference presents non-negligible impacts on the cation transfer number. Under the weak temperature impact condition, the cation transfer number stays nearly unchanged, especially at low concentration differences.

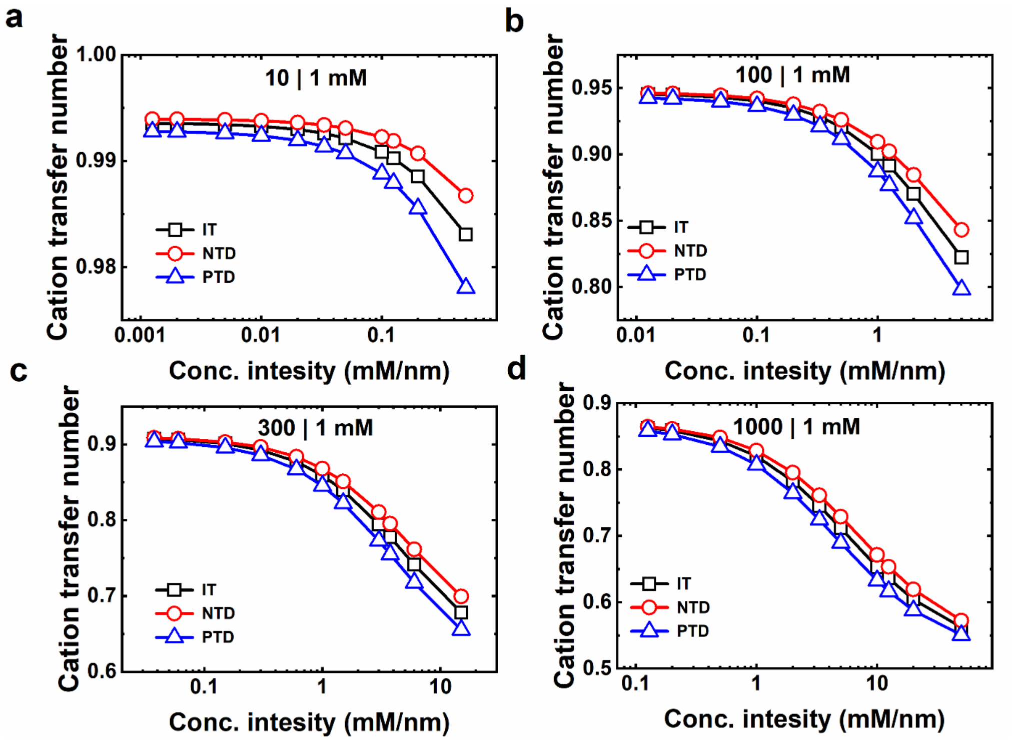

Figure S8. The cation transfer number vs transmembrane concentration intensities under different temperature differences, where IT, PTD, and NTD represent the isothermal conditions (T_L=T_R=298 K), positive temperature difference (T_L=318 K, T_R=298 K) and negative temperature difference (T_L=298 K, T_R=318 K). (a) 10|1 mM, (b) 100|1 mM, (c) 300|1 mM, and (d) 1000|1 mM. The transmembrane concentration intensity is calculated as the concentration difference dividing the nanopore length.

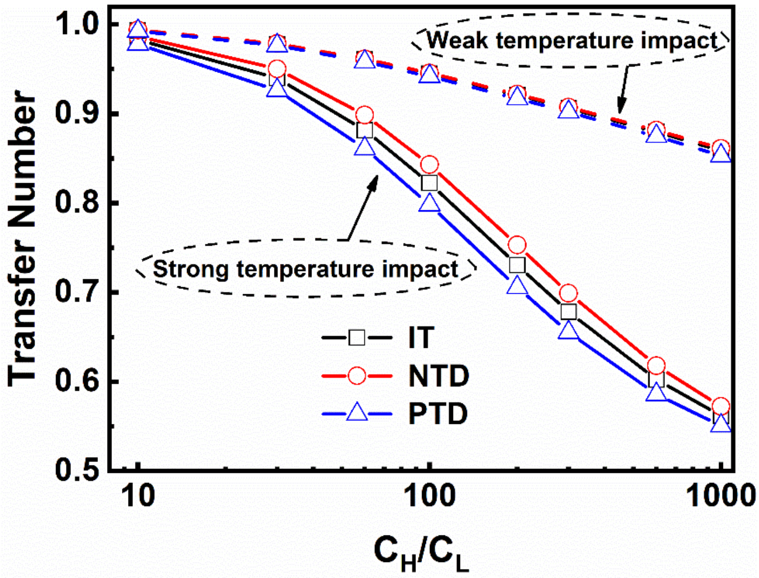

Figure S9. Cation selectivity of nanochannels under different concentration differences, where IT, PTD, and NTD represent the isothermal conditions (T_L=T_R=298 K), positive temperature difference (T_L=318 K, T_R=298 K) and negative temperature difference (T_L=298 K, T_R=318 K). The length of the selected nanochannels is 20nm and 5000nm, under the strong and weak temperature impact conditions, respectively. In the calculation, the concentration difference is 1000-fold.

9. Concentration profiles at long channel lengths

Figure S10 shows the axial ion concentration profiles under different temperature differences. The EDL thickness can be represented by the Debye length λ,which is impacted by ion concentration, $\lambda \propto 1/\sqrt{C}$, where C is the local concentration. Larger concentration leads to less value of the Debye length, resulting in weakened EDL overlapping degree. With a negative temperature difference applied, the diffusion coefficient increases along the diffusion direction. The ion diffusion is enhanced by the ionic thermal up-diffusion, and ion concentrations are reduced in the nanochannel, which contributes to the EDL overlapping, leading to improved selectivity. When a positive temperature applied, ion concentrations are augmented in the nanochannel, resulting in worsened EDL overlapping and a degraded ion selectivity. For low concentration difference, significant EDL overlapping exists in the entire channel. For high concentration difference, significant EDL overlapping only exists near the exit section. Long channel length weakens the transmembrane temperature gradient, and equalizes the solution temperature in the effective channel section and that of the LC reservoir.

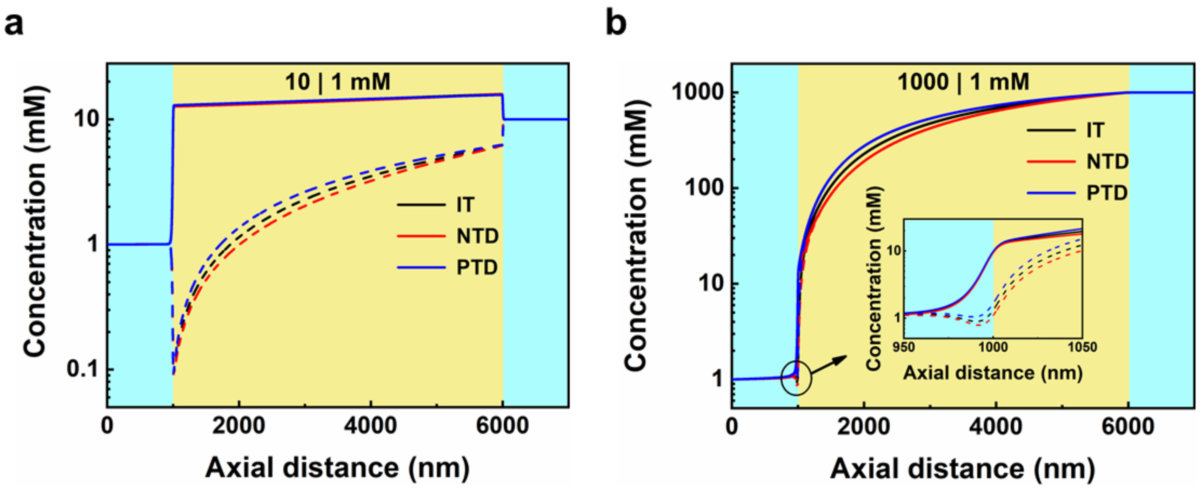

Figure S10. Axial ion concentration profiles under different temperature differences, where IT, PTD, and NTD represent the isothermal conditions (T_L=T_R=298 K), positive temperature difference (T_L=318 K, T_R=298 K) and negative temperature difference (T_L=298 K, T_R=318 K). In the calculation, the length of the nanochannel is 5000nm.

10. Membrane potential

Figure S11 shows the membrane potential vs transmembrane concentration intensities under different temperature differences at high concentration difference (1000-fold). At low concentration intensities, the cation transfer number exhibits slight difference under varied temperature differences. And the ICP is hindered and does not present obvious difference under varied temperature differences. The membrane potential is mainly determined by the temperature in the significant EDL overlapping section. For high concentration differences, significant EDL overlapping only exists near the exit section. Long channel length weakens the transmembrane temperature gradient, and equalizes the solution temperature in the effective channel section and that of the LC reservoir. At this situation the membrane potential is only determined by the temperature at the LC sided.

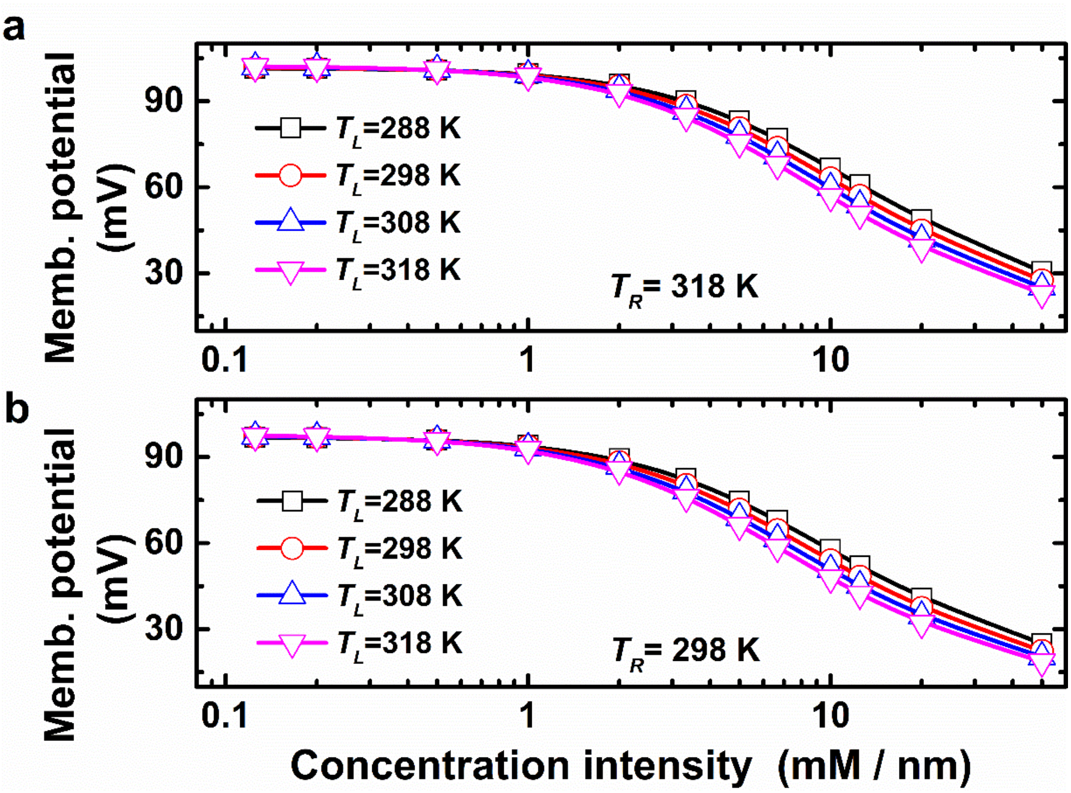

Figure S11. The membrane potential vs transmembrane concentration intensities under different temperature differences. In the calculation, the applied concertation difference is 1000-fold. And the transmembrane concentration intensity is calculated as the concentration difference dividing the nanopore length.

11. Ion concentration polarization

Local ion concentration near the high/low concentration end is presented in Figure S12. The effective ion concentration at low concentration side is higher than the bulk value while at the high-concentration side the effective ion concentration is lower with the respect to the bulk. Under high transmembrane concentration intensities, the ICP is much obvious, which undermines the effective transmembrane concentration difference. And the temperature difference significantly impacts the local ion concentration. A negative temperature difference hinders the ICP at the low-concentration side and contributes to the ICP at the high concentration side. A positive temperature difference decreases the ICP degree at the high concentration side and increases the ICP at the low concentration side. The deviation in local concentration is much prominent at the low concentration end. At low transmembrane concentration intensities, the low concentration at the low/high concentration end presents no obvious difference under varied temperature differences.

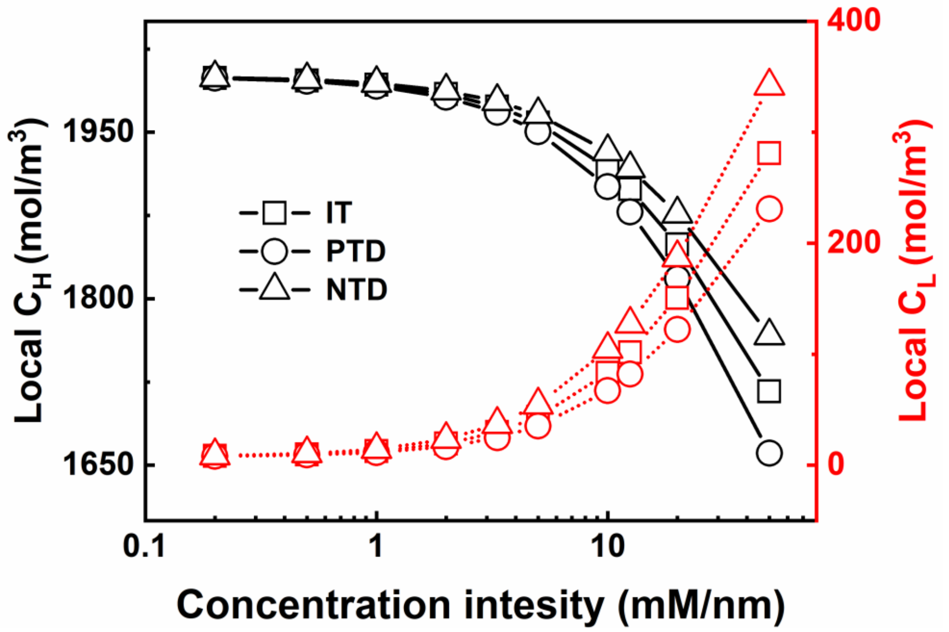

Figure S12. Local ion concentration at the high/low concentration end, where IT, PTD, and NTD represent the isothermal conditions (T_L=T_R=298 K), positive temperature difference (T_L=318 K, T_R=298 K) and negative temperature difference (T_L=298 K, T_R=318 K). These two parameters highly depend on the transmembrane concentration intensities and temperature differences. Negative temperature difference depresses C_L while promotes C_H from their bulk values, and the former acts much significantly. In the calculation, the concentration difference is 1000-fold. The transmembrane concentration intensity is calculated as the concentration difference dividing the nanopore length.

12. Impacts of surface charge density

As shown in Figure S13, under different surface charge densities, a negative temperature difference improves the electric power at high and low transmembrane concentration intensities. The behavior of the extracted power under the positive temperature difference exhibits strong dependence of the transmembrane concentration intensity. At low transmembrane concentration intensities, a positive temperature difference contributes to the power output, which decreases the power at high transmembrane concentration intensities. If the temperature-depended surface charge density considered, in our calculation, the average temperature charge is about 10 K, the impact of the temperature on the surface charge density is rather small in some situations [14], we have also calculated the enhancement factor, by considering the temperature impacted surface charge density by deviating ±4% from that under the isothermal conditions, as shown in Figure S14. We can see that the same phenomenon is observed as that obtained under the constant surface charge density.

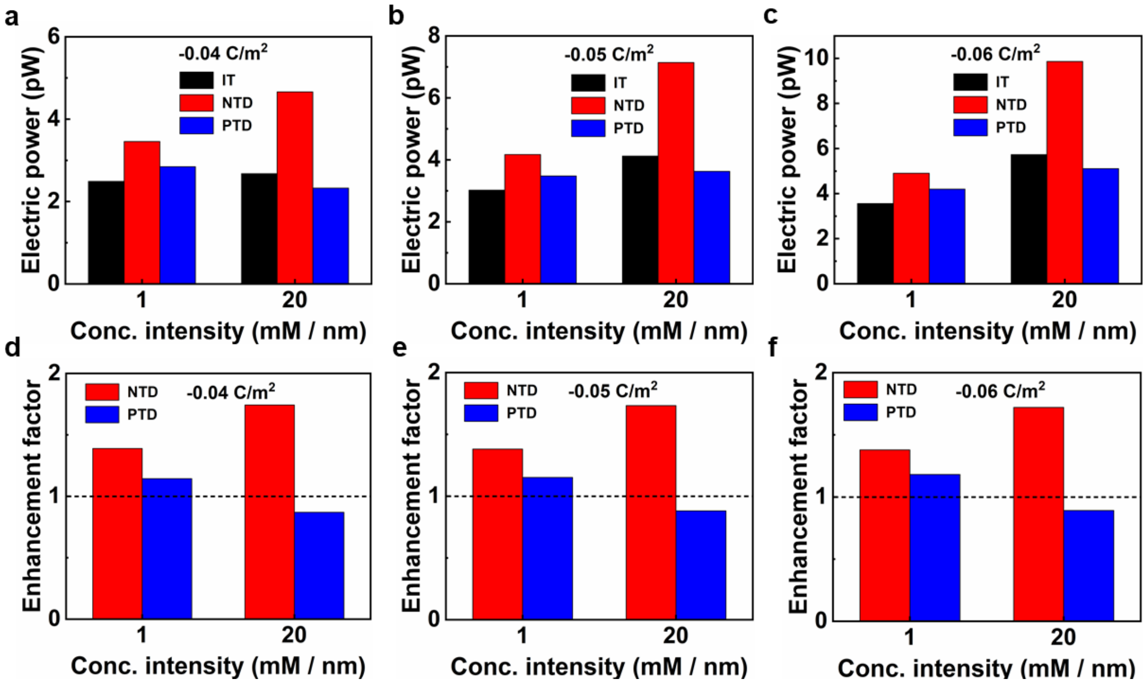

Figure S13. The electric power and enhancement factor under different surface charge densities and high/low transmembrane concentration intensities, where IT, PTD, and NTD represent the isothermal conditions (T_L=T_R=298 K), positive temperature conditions (T_L=318 K, T_R=298 K) and negative temperature conditions (T_L=298 K, T_R=318 K). Here the enhancement factor is defined as the power under the IT condition divided by that under the NTD/PTD.

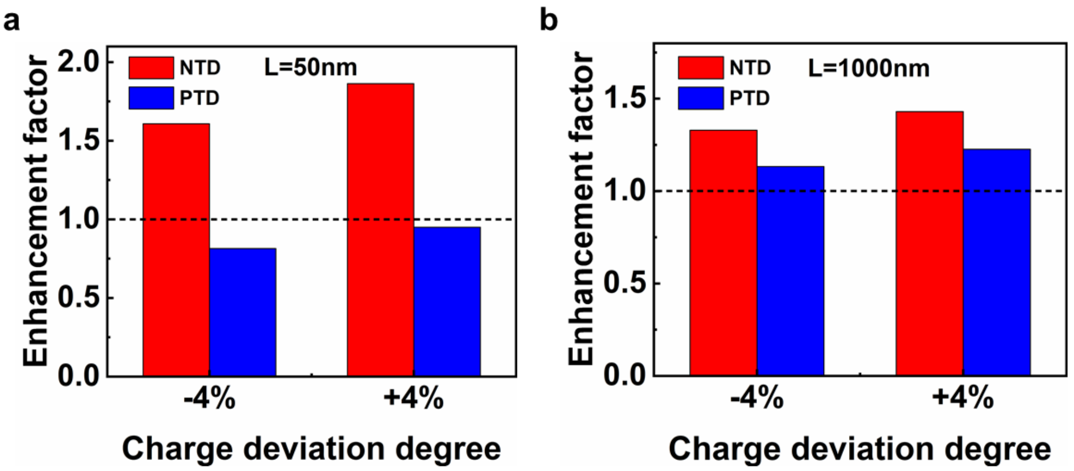

Figure S14. Enhancement factor under slightly deviated surface charge densities and high/low transmembrane concentration intensities, where IT, PTD, and NTD represent the isothermal conditions (T_L=T_R=298 K), positive temperature conditions (T_L=318 K, T_R=298 K) and negative temperature conditions (T_L=298K, T_R=318 K).

13. Impacts of viscous dissipation

We calculate the osmotic current with/without viscous dissipation under different temperature differences for a short channel (L=20 nm) at a concentration difference of 300-fold, as shown in Figure S15. The osmotic current with/without viscous dissipation almost coincides with each other. Therefore, the neglection of the viscous dissipation in the energy conservation equation in present study is justified.

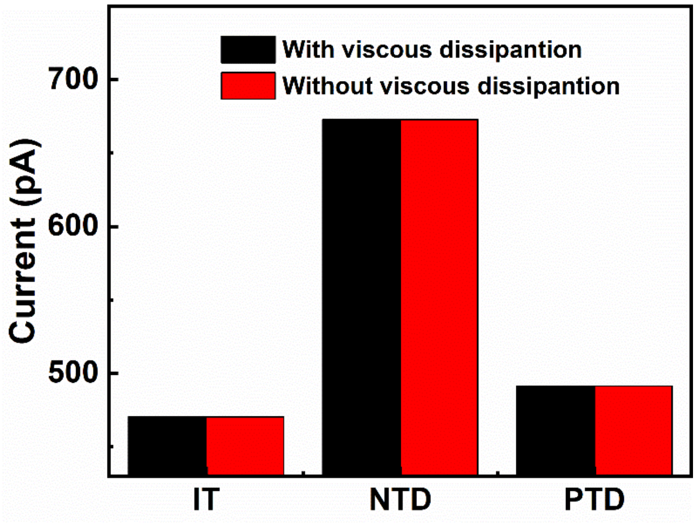

Figure S15. Osmotic current with/without viscous dissipation under different temperature differences for a short channel (L=20 nm). IT, PTD, and NTD represent the isothermal conditions (T_L=T_R=298 K), positive temperature conditions (T_L=318 K, T_R=298 K) and negative temperature conditions (T_L=298 K, T_R=318 K). In the calculation, the concentration difference is 300-fold.

14. Energy efficiency

The energy efficiency with or without considering the transmembrane heat transfer under different transmembrane concentration intensities and temperature differences are analyzed in Figure S16. Based on Equation (S13), the input Gibbs free energy is much decreased under a negative temperature difference, leading to enhanced power output and energy conversion efficiency simultaneously. The positive temperature difference decreases the membrane potential, deteriorates the ion selectivity, and augments Gibbs free energy consumed, resulting in lowered energy efficiency. At a concentration density of 1 mM/nm, the energy efficiency of IT, NTD, and PTD are 18.17%, 19.69% and 16.24%, respectively. Compared to the energy efficiency under the isothermal condition, it is augmented by 8.3% under the negative temperature difference and is decreased by 10.6% under the positive temperature difference. At a concentration density of 20 mM/nm, the energy efficiency of IT, NTD, and PTD are 2.36%, 3.24% and 1.6%, respectively. Compared to the energy efficiency under the isothermal condition, it is augmented by 37.3% under the negative temperature difference and is decreased by 32.2% under the positive temperature difference. If the heat used to heat the salt solutions considered (Equation (S15)), the energy efficiency will be significantly decreased. Therefore, we can use waste heat to construct a negative transmembrane temperature difference, thus to improve its efficiency.

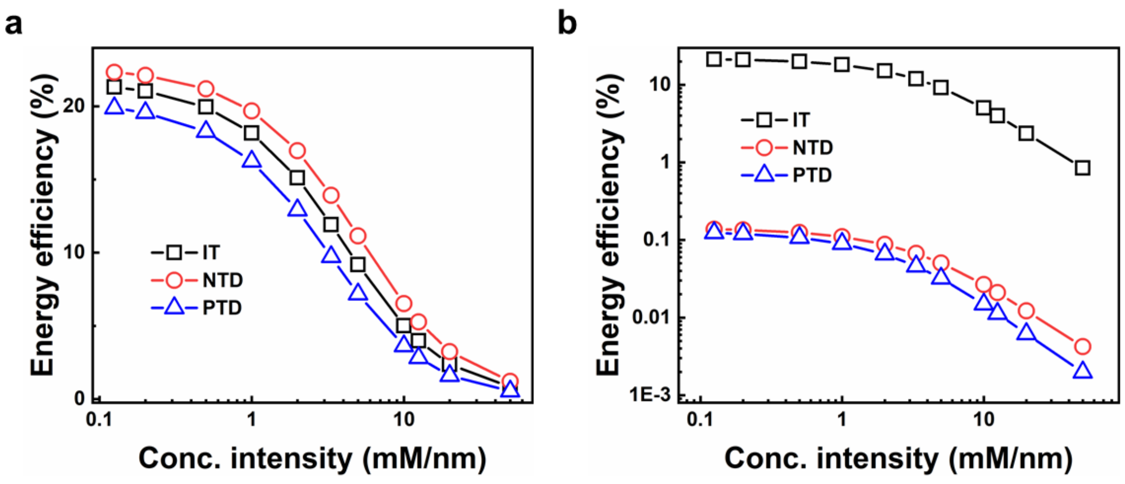

Figure S16. The energy efficiency with (a) or without (b) considering the transmembrane heat transfer under different transmembrane concentration intensities and temperature differences, where IT, PTD, and NTD represent the isothermal conditions (T_L=T_R=298 K), positive temperature difference (T_L=318 K, T_R=298 K) and negative temperature difference (T_L=298 K, T_R=318 K). In the calculation, the concentration difference is 1000-fold. The transmembrane concentration intensity is calculated as the concentration difference dividing the nanopore length.

15. References

[1] W. Guo, L. Cao, J. Xia, F. Q. Nie, W. Ma, J. Xue, Y. Song, D. Zhu, Y. Wang and L. Jiang, Adv. Funct. Mater. 20 (2010) 1339.

[2] D. A. Aikens, J Chem. Educ. 60 (2004) 669.

[3] R. Long, Z. Kuang, Z. Liu, W. Liu, Phys. Chem. Chem. Phys. 20 (2018) 7295.

[4] D. Vigolo, S. Buzzaccaro and R. Piazza, Langmuir 26 (2010) 7792.

[5] B. T. Huang, M. Roger, M. Bonetti, T. J. Salez, C. Wiertelgasquet, E. Dubois, R. C. Gomes, G. Demouchy, G. Mériguet and V. Peyre, J. Chem. Phys. 143 (2015) 054902.

[6] A. Würger, Phys. Rev. Lett. 101 (2008) 108302.

[7] A.W. Jeffery, M.B. Anne, G.H.L. Rob, J. Phys. Cond. Matter, 28 (2016) 114002.

[8] B. B. Owen, R. C. Miller, C. E. Milner and H. L. Cogan, J. Phys. Chem. 65 (1961) 2065.

[9] S. Tseng, J. Y. Lin and J. P. Hsu, Anal. Chim. Acta 847 (2014) 80.

[10] S. Tseng, Y. M. Li, C. Y. Lin and J. P. Hsu, Nanoscale 8 (2016) 2350.

[11] J. P. Hsu, Y. H. Tai, L. H. Yeh and S. Tseng, Langmuir 28 (2012) 1013.

[12] A. Siria, P. Poncharal, A. L. Biance, R. Fulcrand, X. Blase, S. T. Purcell and L. Bocquet, Nature 494 (2013) 455.

[13] C. Y. Lin, F. Chen, L. H. Yeh, and J. P. Hsu, Phys. Chem. Chem. Phys. 18 (2016) 30160.

[14] M. Taghipoor, A. Bertsch, P. Renaud, Acs Nano, 9 (2015) 4563-4571.
